# Long-Term Outcomes and Sites of Failure in Locally Advanced, Cervical Cancer Patients Treated by Concurrent Chemoradiation with or without Adjuvant Chemotherapy: ACTLACC Trial

**DOI:** 10.31557/APJCP.2021.22.9.2977

**Published:** 2021-09

**Authors:** Chokaew Tovanabutra, Tussawan Asakij, Kanisa Rongsriyam, Siriwan Tangjitgamol, Ekkasit Tharavichitkul, Jirasak Sukhaboon, Lieutenant Col. Apiradee Kridakara, Kannika Paengchit, Jakkapan Khunnarong, Thiti Atjimakul, Piyawan Pariyawateekul, Prapai Tanprasert, Tharathorn Tungkasamit, Vichan Lorvidhaya

**Affiliations:** 1 *Radiation Oncology Section, Chonburi Cancer Hospital, Thailand. *; 2 *Radiation Oncology Section, Lampang Cancer Hospital, Thailand. *; 3 *Department of Radiology, Faculty of Medicine Vajira Hospital, Navamindradhiraj University, Thailand. *; 4 *Department of Obstetrics and Gynecology, Faculty of Medicine Vajira Hospital, Navamindradhiraj, University, Thailand. *; 5 *Women’s Health Center, MedPark Hospital, Bangkok, Thailand. *; 6 *Department of Radiology, Faculty of Medicine Chiang Mai University, Thailand. *; 7 *Radiation Oncology Section, Lopburi Cancer Hospital, Thailand. *; 8 *Radiation Oncology Section, Bhumibol Adulyadej Hospital, Thailand. *; 9 *Gynecologic Oncology section, Lampang Cancer Hospital, Thailand. *; 10 *Department of Obstetrics and Gynecology, Prince of Songkla University, Thailand. *; 11 *Obstetrics and Gynecology Section, Bhumibol Adulyadej Hospital, Thailand. *; 12 *Obstetrics and Gynecology Section, Rajburi Hospital, Thailand. *; 13 *Radiation Oncology section, Udonthani Cancer Hospital, Thailand. *

**Keywords:** Locally advanced cervical cancer, concurrent chemoradiation therapy, adjuvant chemotherapy

## Abstract

**Objectives:**

To evaluate sites of failure and long-term survival outcomes of locally advanced stage cervical cancer patients who had standard concurrent chemo-radiation (CCRT) versus those along with adjuvant chemotherapy (ACT) after CCRT.

**Methods:**

Patients aged 18–70 years who had FIGO stage IIB-IVA without para-aortic lymph node enlargement (excluding by International Federation of Gynecology and Obstetrics (FIGO) 2018 stage IIIC2r), The Eastern Cooperative Oncology Group (ECOG) scores 0–2, and non-aggressive histopathology were randomized to have CCRT with weekly cisplatin followed by observation (arm A) or ACT with paclitaxel plus carboplatin every 4 weeks for 3 cycles (arm B).

**Results:**

From 2015-2017, 259 patients were evaluated. The majority of patients were in stage II and had squamous cell carcinoma with a median tumor size of 5 cm. After the median follow-up of 40.87 months, 17.1% of the patients in arm A and 12.3% of the patients in arm B experienced recurrences (p=0.280). Adding all events of failure (persistence/progression/recurrence), treatment failures tended to be lower in arm A than in arm B: 13.2 versus 21.5 % for loco-regional failure (p = 0.076) and 3.9 versus 6.9% for loco-regional failure and systemic failure (p = 0.278). On the other hand, systemic failure tended to be higher in arm A than in arm B: 13.2% versus 6.9% (p =0.094). The 5-year progression-free survival and 5-year overall survival of patients in both arms were not significantly different.

**Conclusions:**

ACT with paclitaxel plus carboplatin after CCRT did not improve response or survival of patients compared to CCRT alone. Although systemic failure tended to be lower in patients who had ACT after CCRT than those who had only CCRT, loco-regional failure with or without systemic failure tended to be higher. However, all of these differences were not statistically significant.

## Introduction

Cervical cancer is a global health problem of women, with an average age standardized incidence rate of 13.1 per 100,000 women and a death rate of 6.9 per 100,000 women (Ferlay et al., 2019). The problem is especially encountered in developing countries including Thailand (Ministry of Public Health and Ministry of Education, 2015). Although cervical cancer was the second most common female cancer after breast cancer in our country, the proportion of death rate to incidence rate of cervical cancer was much higher than that of breast cancer. The respective average age standardized incidence and death rates per 100,000 women were 16.2 and 9.0 (slightly more than half) for cervical cancer compared to 35.7 and 10.9 (nearly one third) for breast cancer (Ministry of Public Health and Ministry of Education, 2015). This is probably due to a suboptimal screening coverage of the target population, leading to a high proportion of locally advanced and advanced stage diseases at diagnosis (Khuhaprema et al., 2012).

Although concurrent chemoradiation therapy (CCRT) is a current standard treatment for locally advanced cervical cancer (LACC), high rates of local and distant failures (17% and 18% respectively) and unsatisfactory survival outcomes (approximate 5-year overall survival (OS) rate of only 60%) were still encountered (Chemoradiotherapy for cervical cancer meta-analysis collaboration, 2008). Hence, other treatments were added to CCRT i.e. combining targeted agents with chemotherapy, giving neoadjuvant (NACT) or adjuvant chemotherapy (ACT) prior to or after CCRT. 

Regarding adjuvant chemotherapy after CCRT, many prospective phase II studies showed improved response rates and high 80–90% survival rates with adjuvant or consolidation chemotherapy (Vrdoljak et al.2006; Domingo et al., 2009; Choi et al., 2010; Zhang et al., 2010). However, there were inconsistent data regarding progression-free survival (PFS) and disease-free survival (DFS) from previous randomized controlled trials (RCT) showing either increased survival (Dueñas-González et al., 2011; Tang et al., 2012) or no such benefit from ACT (Lorvidhaya et al., 2003; Veerasan et al., 2007). Without a definite conclusion, our group conducted another RCT comparing CCRT using weekly cisplatin as chemotherapy during radiation versus CCRT (same regimen of chemotherapy) plus ACT (paclitaxel and carboplatin) (Tangjitgamol et al., 2019). Details of primary tumor response and primary failure including persistence and progression at each month of assessment, and the PFS and OS after a median follow-up duration of approximately 2 years were presented in the previous report (Tangjitgamol et al., 2019). The cost-utility of adjuvant chemotherapy was also studied and will be presented elsewhere.

This study aimed to evaluate treatment outcomes after long-term follow-up in terms of recurrence rate, sites of failure, and survival of patients who had CCRT alone or ACT after CCRT. The outcomes according to the new International Federation of Gynecology and Obstetrics (FIGO, 2018) staging (Bhatla et al., 2019) were also studied. 

## Materials and Methods

The trial was a collaboration among 11 institutions in Thailand. The protocol was approved by the National Central Research Ethics Committee (COA-CREC 002/2013) and was registered under the TCTR (TCTR 20140106001) and the clinicalTrials.gov (NCT02036164). 

*Sample Size*

Details of sample size calculation were described in our previous report (Tangjitgamol et al., 2019). In brief, the sample size was based on the study of Choi et al., (2010) who found a 15% improvement of PFS in patients who had ACT after CCRT (70%) compared to CCRT alone (55%). With a two-tailed hypothesis and criteria of 0.05 (alpha) to determine the significance, 220 subjects in each group were required. We primarily set a statistical power of 90%, so 500 subjects were planned (250 subjects in each arm) with an estimated 158 total number of events. When an interim analysis (where 271 patients were enrolled) showed no definite benefit of ACT, further patient recruitment was stopped as planned due to the futility of further chemotherapy as detailed in the previous report (Tangjitgamol et al., 2019).

*Inclusion Criteria*

The inclusion criteria were women aged 18 to 70 years who had: newly diagnosed cervical cancer (FIGO 2009 stage IIB-IVA), histopathology of squamous cell carcinoma, adenocarcinoma, or adeno-squamous carcinoma, ECOG performance score of 0–2, adequate bone marrow reserve, and adequate hepatic and renal functions. Patients who had para-aortic lymph > 1 cm or suspected cancer metastasis following a CT scan (stage IIICr2 by FIGO 2018), had received other experimental drugs in the past 30 days or had uncontrolled medical illness i.e. pre-existing neuropathy or HIV infection were excluded. All patients signed informed consent forms to participate in the study.

*Randomization and Treatment*

**Figure 1:**
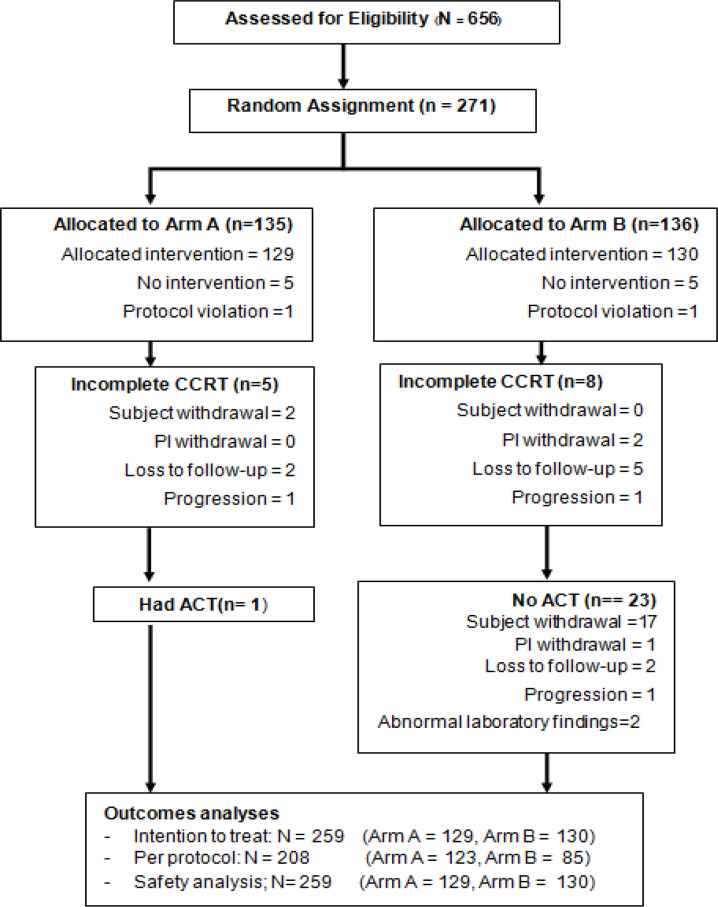
CONSORT Diagram of Study Design. Arm A received concurrent weekly cisplatin with pelvic radiation therapy and brachytherapy (CCRT). Arm B received concurrent weekly cisplatin with pelvic radiation therapy and brachytherapy, followed by adjuvant chemotherapy (ACT) with paclitaxel and carboplatin for 3 cycles

The study CONSORT diagram is presented in Figure 1. All patients who met all inclusion criteria were randomly assigned to arm A or arm B at week zero using nQuery 7.0 (Stasols, Boston, MA, USA) and stratified by disease stage (IIB vs III to IVA) and histopathology (squamous versus adenocarcinoma or adenosquamous carcinoma) by mixed block randomization. The block random allocation sequence was obtained using a central computerized-generated randomization system. The enrollment and arm of treatment assignment were done using the trial website (http://actlacc.thaimedresnet.org). 

The principal investigators in each hospital provided treatment according to the protocol. Before the project was launched, the investigators from all participating institutions conferred to standardize the radiation instrument and techniques, and the details of chemotherapy treatment. The treatment was required to be initiated within 30 days after randomization and was given in each participating hospital. All patients in both the control (arm A) and study arm (arm B) received weekly cisplatin 40 mg/m2 concurrent with pelvic radiation therapy. After CCRT, the patients in arm A underwent surveillance without any additional treatment. Those in arm B, after a 4-week period, had paclitaxel 175 mg/m^2^ IV plus carboplatin AUC 5 IV every 4 weeks for 3 cycles. Details of radiation and chemotherapy treatment, and dose modification were presented in the Data Supplement of the previous report (Tangjitgamol et al., 2019).

*Data Management and Statistical Analysis*

The National Medical Research Network group of Thailand (MedResNet) managed all data records in each participating hospital. The Data Management Unit of MedResNet, managed by the central research coordinators, verified all submitted data. 

Data were analyzed using SPSS statistical software, version 22.0 (IBM Corp., Armonk, NY, USA). Data from subgroup analysis were compared using the Chi square test. Survival data were analyzed using the Kaplan–Meier method and were compared between groups with a log-rank test. P-values < 0.05 were considered statistically significant. Data were analyzed by a modified intention-to-treat analysis, including all patients who had at least initiated treatment according to their randomized arms and per protocol including only those who had actual treatment as specified in the protocol. 

## Results

From January 2015 to June 2017, 271 eligible patients were enrolled and randomly allocated to either arm A (n= 135) or arm B (n= 136). Data collection was suspended in March 2018 for an interim analysis. A total of 129 patients in arm A and 130 patients in arm B received the allocated treatment (Figure 1). 

Detailed basic characteristics of the patients including stage, histopathology, size of tumor, hemoglobin level at baseline, and data of treatment during CCRT and ACT were presented in our previous report (Tangjitgamol et al., 2019). In brief, the majority of patients were in stage II and had squamous cell carcinoma with a median tumor size of 5 cm (range 2–10 cm). 

During the CCRT phase, 5.0% did not complete CCRT (3.9% in arm A and 6.2% in arm B). The median radiation dose, duration of radiotherapy, and cycles of cisplatin, and hemoglobin level during CCRT were approximately equal between both arms. Regarding the ACT intervention, 23.1% did not have ACT as they could not complete CCRT or they declined further treatment or they were lost to follow-up after CCRT completion. The others had ACT for 3 cycles (65.4%), 2 cycles (6.2%) or 1 cycle (5.4%). The most common reasons for ACT discontinuation were prolonged hematologic toxicity or peripheral neuropathy. The reasons for having no or incomplete CCRT or ACT were shown in the Data Supplement of the previous report (Tangjitgamol et al., 2019). One patient in Arm A had ACT and completed 3 cycles of treatment. 

The clinical outcomes of all 259 patients (129 in arm A and 130 in Arm B) were shown by an intention to treat. Details of tumor response and primary failure including persistence and progression at each month of assessment were summarized in the Data Supplement of our previous report (Tangjitgamol et al., 2019). In brief, the persistence rate was lower in arm A (10 patients or 7.8% versus 21 patients or 16.2%, p = 0.037) without significant difference of disease progression (7 patients or 5.4% versus 9 patients or 6.9%, p = 0.617). Recurrences were observed in 38 patients: 22 in arm A (17.1%) versus 16 (12.3%) in arm B (p = 0.280). A summary of events and the sites of treatment failure were studied in detail. Each outcome of treatment (complete response without evidence of disease versus failure with any events of persistence, progression, or recurrence) according to the arm of treatment is shown in Table 1. 

Treatment outcomes were studied according to the characteristic features of the patients and their disease by treatment arm. The recurrence rate was significantly lower in stage IIIC1r patients in arm B: 12.1% versus 37.9% (p = 0.018). There were no influences on any treatment outcomes of other characteristic features of the patients, their diseases, and treatments received (Table 1). 

**Figure 2:**
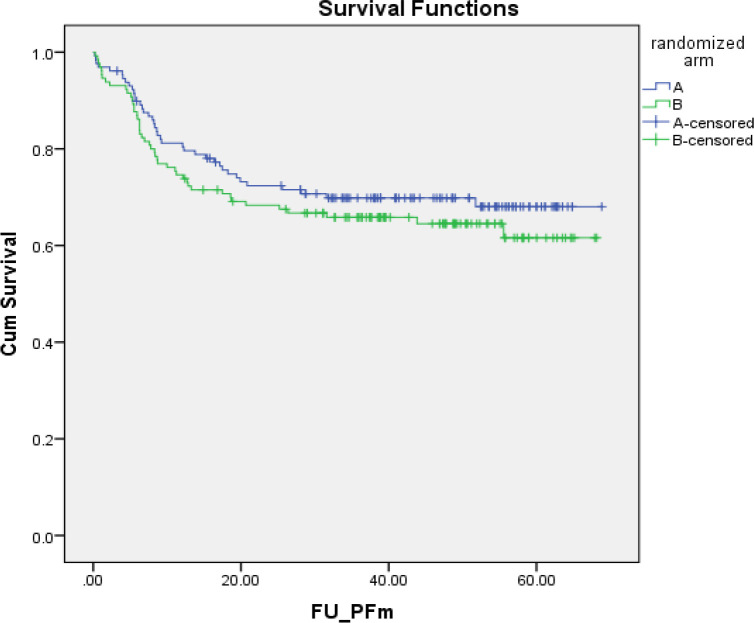
PFS of Cervical Cancer Patients in arm A (n= 129, concurrent chemoradiation) and arm B (n= 130, concurrent chemoradiation plus adjuvant chemotherapy) 5-year PFS: Arm A = 65.9% (95% CI, 57.7–74.1%) versus arm B = 61.6% (95% CI, 51.8–71.4%). Hazard ratio for PFS = 1.22 (95% CI,0.80–1.87; p = 0.354)

**Figure 3:**
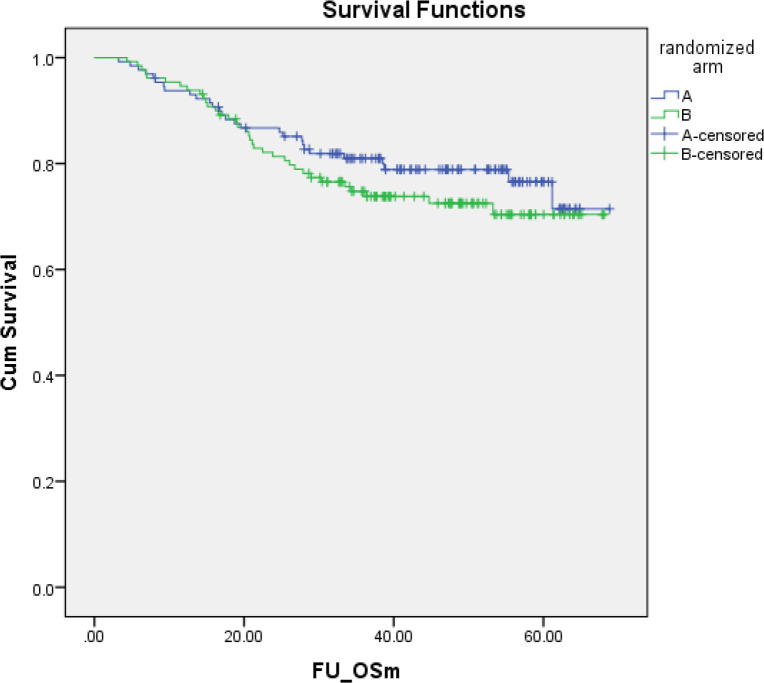
Overall Survival of Cervical Cancer Patients in Arm A (n= 129, concurrent chemoradiation) and arm B (n =130, concurrent chemoradiation plus adjuvant chemotherapy) 5-year OS: Arm A = 76.5% (95% CI, 68.1–84.9%) versus Arm B = 70.4% (95% CI, 61.2-79.2%). Hazard ratio for OS = 1.27 (95% CI,0.76–2.10; p = 0.339)

With a median follow-up of 40.9 months (range 3.2–69.8 months), the median PFS was 37.6 months (range 0.2–68.8 months). The 5-year PFSs of the patients in both arms were not statistically significantly different: 69.8% (95% CI, 61.8–77.8%) in arm A versus 68.0% (95%CI, 59.4–76.6%) in arm B (Figure2). The hazard ratio for PFS was 1.22 (95% CI, 0.80-1.87; p = 0.354). Overall, 63 had died: 28 (21.7%) in arm A versus 35 (26.9%) in arm B (p = 0.328). The median OS was 40.9 months (range 3.23–68.8 months). The 5-year OSs of the patients in both arms were not statistically significantly different: 76.5% (95% CI, 68.1–84.9%) in arm A versus 70.4% (95% CI, 61.2–79.2%) in arm B (Figure 3). The hazard ratio for OS was 1.27 (95% CI, 0.76–2.10; p = 0.339). 

Regarding the sites of failure, loco-regional failure with or without systemic failure tended to be higher in arm B than in arm A. On the other hand, systemic failure tended to be lower in arm B than in arm A. However, all of the differences were not statistically significant (Table 2). The sites of failure according to the arm of treatment were further studied by the clinical features of the patients (Extra Table 2). Although systemic failure rates were lower in most subgroups among the patients who had ACT, this was significant for only those who were aged over 40 (Tables 3 and 4).

Among 85 patients who had failure after treatment (32.8%), 39 patients (30.2%) were in arm A and 46 patients (35.4%) were in arm B. There werde 103 sites of systemic failure. The most common sites in order of frequency were pelvic cavity (48.5%), para-aortic lymph node (15.5%), bone (8.7%), lung (7.8%), liver (7.8%) and supraclavicular lymph node (4.9%). The sites of failure by treatment arm are shown in Table 3. 

## Discussion

Concurrent radiation with platinum-based chemotherapy is the current standard treatment for LACC (Morris et al., 1999; Rose et al., 1999; Eifel et al., 2004). However, 15–61% of patients still suffered from treatment failure in the first 2 years after treatment (Tangjitgamol et al., 2014). To overcome treatment failure, additional chemotherapy after CCRT was used an option aiming to control residual disease outside the radiation field. However, the results from previous studies on the benefit of adjuvant chemotherapy following CCRT in LACC were inconsistent (Lorvidhaya et al., 2003; Veerasan et al., 2007; Dueñas-González et al., 2011), with inconclusive evidence from a systematic review (Tangjitgamol et al., 2014). These data encouraged our group to investigate the role of ACT in LACC by conducting a trial comparing CCRT or CCRT followed by ACT (Tangjitgamol et al., 2019). Paclitaxel and carboplatin for 3 cycles were used as the ACT after completion of CCRT in the study arm. 

The results from our previous analysis were reported after a median follow-up of 27.4 months (range 3.2–49.0 months). No significant differences of persistence or progression were found in both arms. Although the recurrence was higher in the patients who had only CCRT than those having ACT (approximately 16% versus 11%), the difference was not statistically significant (p = 0.123). Regarding the sites of failure, similar rates of loco-regional failure were observed (3% in both arms). Nevertheless, systemic failures were significantly lower with the use of ACT than CCRT alone, approximately 5% versus 10% (p = 0.029). We could not demonstrate improvement of PFS or OS after ACT. On the contrary, patients in arm B had approximately 3% lower 3-year PFS and 11% lower 3-year OS than those who had only standard CCRT. The hazard ratios (HRs) were 1.26 (p = 0.293) for PFS and 1.42 (p = 0. 221) for OS. 

After a longer follow-up period of nearly 70 months, no significant differences of recurrence rates in both arms were demonstrated. However, the possible benefit of ACT in stage IIIC1r should be emphasized, with the finding of a significant reduction of recurrence rate from 38% with CCRT to 12% with ACT (p = 0.018). 

Regarding survival, the PFS and OS of the patients in both arms were still not significantly different after a long follow-up. The 5-year PFSs and 5-year OSs of the patients who had ACT after CCRT compared to CCRT alone were not significantly different: HR 1.22 (p = 0.354) for PFS and HR 1.27 (p = 0.339) for OS. Our negative findings on survivals were consistent with 2 previous trials from Thailand (Lorvidhaya et al., 2003; Veerasan et al., 2007) and were different from other trials which showed improvement of PFS and OS with the use of ACT after CCRT (Dueñas-González et al., 2011; Tang et al., 2012). A summary of data from previous trials and our study which compared survival LACC patients who received CCRT or CCRT plus ACT is shown in Table 4. A few possible reasons for the different survival rates of those who received ACT in each trial were discussed in detail in our previous report (Tangjitgamol et al., 2019). In brief, there was an imbalance of treatment between the comparative groups in the 2 trials which demonstrated improved survival in the patients who had received ACT (Dueñas-González et al., 2011; Tang et al., 2012). In the trial by Dueñas-González et al., patients in the ACT group had combination chemotherapy (cisplatin/gemcitabine) during CCRT before continuation of the same regimen for 2 more cycles whereas only cisplatin was used concurrently with radiation in the CCRT only group (Dueñas-González et al., 2011). In a successful trial of Tang et al., one cycle of neoadjuvant chemotherapy was given prior to CCRT and adjuvant chemotherapy (Tang et al., 2012). The disparity of treatment might have falsely suggested better efficacy of chemotherapy given in an adjuvant setting. On the other hand, the negative finding of survival in the trial of Lorvidhaya et al. might be questioned because the compliance of oral chemotherapy used as adjuvant treatment was not obtained (Lorvidhaya et al., 2003). We did not find any obvious reason for our negative finding on survival. Many possible causes were proposed, in detail, in our previous report (Tangjitgamol et al., 2019). First, although the characteristic features of the patients and their diseases were well balanced between the 2 treatment groups, we postulated that there might be some insignificant or unrecognized imbalance of the diseases’ characteristic features of the patients who had only CCRT or CCRT and ACT, for example, the presence of stage IVA only in arm B (CCRT/ACT) or initial tumor volume which may give a better prognostic value than the maximal tumor dimension used. This might have resulted in a higher rate of persistence/progressive disease in the ACT group. Second, the 3 cycles of adjuvant chemotherapy may be inadequate to give a clinical benefit of ACT. This was supported by the general practice in advanced ovarian cancer that at least 6 cycles of ACT with or without maintenance treatment are required. Data from a recent retrospective study which found improved survival with 3–6 cycles of paclitaxel/carboplatin given as ACT should further support the idea that more cycles of adjuvant treatment are needed to show clinical efficacy (Yavas et al., 2019).

Regarding the sites of failure, we could not find any differences between those of the patients who had ACT after CCRT versus CCRT alone. Patients who had ACT after CCRT (arm B) tended to have higher loco-regional failure in the pelvis (including persistence and recurrence) (22% versus 13%; p = 0.076) but lower failure in distant sites (7% versus 13%; p = 0.094). Among previous studies and trials which reported the sites of failure in patients who had CCRT with or without ACT, data were in the same direction as regards findings on survival. Of the 2 trials which did not find a survival benefit of ACT (Lorvidhaya et al., 2003; this study), neither found any significant reduction of failure rates at any sites. On the other hand, all studies which showed the survival benefit of ACT also demonstrated reduction of both loco-regional and systemic failures with the use of ACT (Table 5).

The theoretical reduction of failure by ACT after CCRT was supported by findings from previous studies which showed the survival benefit of CCRT/ACT over CCRT alone (Dueñas-González et al., 2011; Tang et al., 2012; Yavas et al., 2019). The differences were significant in 2 trials for loco-regional failure (Tang et al., 2012; Yavas et al., 2019) and all 3 trials for systemic failure (Dueñas-González et al., 2011; Tang et al., 2012; Yavas et al., 2019). The reduction of systemic failure by ACT was also found in our study, but without statistical significance. This might be due partly to the small number of patients experiencing this event in our study. The higher loco-regional failure (including disease persistence/progression) found in our study was unexpected. We can explain this with the same reasoning as described above regarding the unrecognized worse prognostic features in the CCRT/ACT group. Another observation was a higher percentage of incomplete CCRT treatment among patients in the CCRT/ACT group (most were from subject withdrawal rather than toxicity). Although the drop-out rates were not statistically significant, they might influence the rate of loco-regional failure. Nevertheless, with a reduction of systemic failure with ACT, this information may suggest some advantages of ACT. A systematic review and meta- analysis may provide evidence-based data on this issue. 

In conclusion, no significant benefit of paclitaxel with carboplatin given for 3 cycles after a standard concurrent chemoradiation treatment for locally advanced cervical cancer was demonstrated. We were aware of a few limitations in our study which did not allow us to make any general conclusion regarding the role of ACT for LACC. One major limitation was poor compliance of the patients in the trial. From our previous publication, although the discontinuation rates during CCRT were not significantly different between the 2 treatment arms, 18% of all patients who were randomized to have ACT did not have any ACT (or 19% among those who had complete CCRT) (Tangjitgamol et al., 2019).

With inconsistent data from available literature regarding the role of ACT in LACC, other options to improve treatment outcomes should be considered. Some findings in our study and the literature review may have implications for clinical practice or future research. First, the pattern of failure in our cohort showed a higher pelvic failure rate in comparison to other studies. Hence, more aggressive treatment during (or prior to) CCRT should be given for all patients with LACC. This proposal is supported by findings from the 2 positive trials that neoadjuvant chemotherapy prior to or a doublet regimen during CCRT was given for the patients in the ACT group (Dueñas-González et al., 2011; Tang et al., 2012) and a systematic review and meta-analysis which found significantly improved PFS (HR 0.78, p = 0.01) and OS (HR 0.75, p = 0.01) with a doublet chemotherapy regimen, over a single drug (Ma et al., 2019). However, these survival advantages with a doublet regimen must be balanced with the higher risk of hematologic toxicity, especially thrombocytopenia (Dueñas-González et al., 2011; Tang et al., 2012; Ma et al., 2019). Second, more cycles of ACT should be tested in future studies or in clinical applications so that their efficacy might be better demonstrated. as in advanced ovarian cancer or as found in one retrospective study of ACT in LACC which found a survival benefit with ACT of up to 6 cycles (Yavas et al., 2019). Third, based on a significant reduction of recurrences with ACT, particularly stage IIIC1r demonstrated in our study, this may be used as a selective inclusion criterion in future studies with a larger cohort to confirm this positive finding from our study. Finally, for future clinical trials involving LACC, adaptive randomization in favor of standard treatment with CCRT (with modification of the chemotherapy regimen, and preferably a doublet regimen) should be considered.

**Table 1:** Outcomes of Cervical Cancer Patients According to Clinical Features and Arm of Treatment

Features of patient	Persistence/Progression			Recurrence		
	Arm A n=17	Arm B n=30	p-value	Arm A n=22	Arm B n=16	p-value
Age						
≤ 40 years, n=43	2 (4.7)	4 (9.3)	0.564	2 (4.7)	6 (13.9)	0.226
> 40 years, n=216	15 (6.9)	26 (12.0)	0.143	20 (9.3)	10 (4.6)	0.091
Histology						
SCC, n=198	13 (6.7)	23 (11.6)	0.076	15 (7.6)	8 (4.0)	0.129
ACA/AS, n=61	4 (6.6)	7 (11.5)	0.289	7 (11.5)	8 (13.1)	0.51
FIGO 2018 stage						
IIB, n=131	6 (4.6)	12 (9.2)	0.2	5 (3.8)	7 (5.3)	0.68
IIIA, n=3	1 (33.3)	-	-	1 (33.3)	-	-
IIIB, n=60	5 (8.3)	5 (8.3)	0.558	5 (8.3)	5 (8.3)	0.338
IIIC1r, n=62	5 (8.1)	11 (17.7)	0.149	11 (17.7)	4 (6.5)	0.018
IVA, n=3	-	2 (66.7)		-	-	
Tumor size						
≤ 4 cm, n=96	4 (4.2)	9 (9.4)	0.158	5 (5.2)	5 (5.2)	0.944
> 4 cm, n=163	13 (8.0)	21 (12.9)	0.114	17 (10.4)	11 (6.7)	0.323
Total radiation dose						
<85 Gy, n=23	4 (17.4)	9 (39.1)	0.645	-	1 (4.3)	0.455
≥85 Gy, n=236	13 (9.6)	21 (8.9)	0.1	22 (9.3)	15 (6.4)	0.327
Total treatment times						
≤ 56 days, n=123	7 (5.7)	15 (12.2)	0.094	9 (7.3)	8 (6.5)	0.822
>56 Days, n=136	10 (7.4)	15 (11.0)	0.325	13 (9.6)	8 (5.9)	0.297
Cisplatin cycle						
<5 cycles, n=67	10 (14.9)	10 (14.9)	0.811	3 (4.5)	1 (1.5)	0.347
≥ 5 cycles, n=192	7 (3.6)	20 (3.6)	0.216	19 (9.9)	15 (7.8)	0.486
Cisplatin total dose						
<200 mg, n=52	7 (13.5)	11 (21.2)	0.573	1 (1.9)	3 (5.8)	0.42
≥200 mg, n=207	10 (4.8)	19 (9.2)	0.062	21 (10.1)	13 (5.3)	0.254

**Table 2:** Sites of Treatment Failure According to Clinical Features of Cervical Cancer and Treatment

Features	Total	Treatment Failure			
		No	Yes		
			Persistence	Progression	Recurrence
Histology					
SCC	198	139 (70.2)	21 (10.6)	15 (7.6)	23 (11.6)
ACA/ AS	61	35 (57.4)	10 (16.4)	1 (1.6)	15 (24.6)
Age group					
≤ 40 years	43	29 (67.4)	4 (9.3)	2 (4.7)	8 (18.6)
> 40 years	216	145 (67.1)	27 (12.5)	14 (6.5)	30 (13.9)
FIGO 2018 stage					
IIB	131	101 (77.1)	11 (8.4)	7 (5.3)	12 (9.2)
IIIA	3	1 (33.3)	-	1 (33.3)	1 (33.3)
IIIB	60	40 (66.7)	8 (13.3)	2 (3.3)	10 (16.7)
IIIC1r	62	31 (50)	11 (17.7)	5 (8.1)	15 (24.2)
IVA	3	1 (33.3)	1 (33.3)	1 (33.3)	-
Tumor size					
≤ 4 cm.	96	73 (76)	7 (7.3)	6 (6.3)	10 (10.4)
> 4 cm.	163	101 (62)	24 (14.7)	10 (6.1)	28 (17.2)
Total radiation dose					
<85 Gy	23	9 (39.1)	10 (43.5)	3 (13)	1 (4.3)
≥85 Gy	236	165 (69.9)	21 (8.9)	13 (5.5)	37 (15.7)
Total treatment times					
≤ 56 days	123	84 (68.3)	16 (13)	6 (4.9)	17 (13.8)
>56 Days	136	90 (66.2)	15 (11)	10 (7.4)	21 (15.4)
Cisplatin cycle					
<5 cycles	67	43 (64.2)	16 (23.9)	4 (6)	4 (6)
≥ 5 cycles	192	131 (68.2)	15 (7.8)	12 (6.3)	34 (17.7)
Cisplatin total dose					
<200 mg	52	30 (57.7)	15 (28.8)	3 (5.8)	4 (7.7)
≥200 mg	207	144 (69.6)	16 (7.7)	13 (6.3)	34 (16.4)

**Table 3:** Sites of Treatment Failure According to Clinical Features of Cervical Cancer and Treatment

Features	Treatment failure			
	Total	Loco-regional	Systemic	Loco-regional and systemic
Age group				
≤ 40 years	14	6 (42.9)	5 (35.7)	3 (21.4)
> 40 years	71	39 (54.9)	21 (29.6)	11 (15.5)
FIGO 2018 stage				
IIB	30	14 (46.7)	12 (40)	4 (13.3)
IIIA	2	1 (50)	1 (50)	-
IIIB	20	10 (50)	4 (20)	6 (30)
IIIC1r	31	19 (61.3)	9 (29)	3 (9.7)
IVA	2	1 (50)	-	1 (50)
Histology				
SCC	59	33 (55.9)	17 (28.8)	9 (15.3)
ACA/ AS	26	12 (46.2)	9 (34.6)	5 (19.2)
Tumor size				
≤ 4 cm.	23	13 (56.5)	7 (30.4)	3 (13)
> 4 cm.	62	32 (51.6)	19 (30.6)	11 (17.7)
Total radiation dose				
Total dose <85 Gy	14	12 (85.7)	-	2 (14.3)
Total dose ≥85 Gy	71	33 (46.5)	26 (36.6)	12 (16.9)
Total treatment times				
≤ 56 days	39	22 (56.4)	10 (25.6)	7 (17.9)
>56 Days	46	23 (50)	16 (34.8)	7 (15.2)
Brachytherapy techniques				
2D Planning	63	31 (49.2)	22 (34.9)	10 (15.9)
3D Planning	22	14 (63.6)	4 (18.2)	4 (18.2)
Cisplatin cycle				
<5 cycles	24	18 (75)	3 (12.5)	3 (12.5)
≥ 5 cycles	61	27 (44.3)	23 (37.7)	11 (18)
Cisplatin total dose				
<200 mg	22	18 (81.8)	2 (9.1)	2 (9.1)
≥200 mg	63	27 (42.9)	24 (38.1)	12 (19)

**Table 4:** Sites of Treatment Failure According to Clinical Features of Cervical Cancer and Arm of Treatment

Features of patient	LR (n=45)			Systemic (n=26)			Systemic and local (n=14)		
	Arm A n=17	Arm B n=28	p-value	Arm A n=17	Arm B n=9	p-value	Arm A n=5	Arm B n=9	p-value
Age									
≤ 40 years, n=43	2 (4.7)	4 (9.3)	0.564	2 (4.7)	3 (7.0)	0.841	-	3 (7.0)	0.11
> 40 years, n=216	15 (6.9)	24 (11.1)	0.098	15 (6.9)	6 (2.8)	0.043	5 (2.3)	6 (2.8)	0.372
Histology									
SCC, n=198	13 (6.6)	20 (10.1)	0.204	11 (5.6)	6 (3.0)	0.19	4 (2.0)	5 (2.5)	0.756
ACA/AS, n=61	4 (6.6)	8 (13.1)	0.176	6 (9.8)	3 (4.9)	0.303	1 (1.6)	4 (6.6)	0.15
FIGO 2018 stage									
IIB, n=131	5 (3.8)	9 (6.9)	0.357	5 (3.8)	7 (5.3)	0.68	1 (0.8)	3 (2.3)	0.364
IIIA, n=3	1 (33.3)	-	-	1 (33.3)	-	-	-	-	-
IIIB, n=60	4 (6.7)	6 (10.0)	0.198	3 (5.0)	1 (1.7)	0.484	3 (5.0)	3 (5.0)	0.663
IIIC1r, n=62	7 (11.3)	12 (19.4)	0.297	8 (12.9)	1 (1.6)	0.639	1 (1.6)	2 (3.2)	0.632
IVA, n=3	-	1 (33.3)	-	-	-	-	-	1 (33.3)	-
Tumor size									
≤ 4 cm, n=96	5 (5.2)	8 (8.3)	0.416	4 (4.2)	3 (3.1)	0.653	-	3 (3.1)	0.085
> 4 cm, n=163	12 (7.4)	20 (12.3)	0.106	13 (8.0)	6 (3.7)	0.093	5 (3.1)	6 (3.7)	0.739
Total radiation dose									
<85 Gy, n=23	4 (17.4)	8 (34.8)	0.879	-	-	-	-	2 (8.7)	0.28
≥85 Gy, n=236	13 (5.5)	20 (8.5)	0.141	17 (7.2)	9 (3.8)	0.127	5 (2.1)	7 (3.0)	0.494
Total treatment times									
≤ 56 days, n=123	7 (5.7)	15 (12.2)	0.217	6 (4.9)	4 (3.3)	0.527	3 (2.4)	4 (3.3)	0.681
>56 Days, n=136	10 (7.4)	13 (9.6)	0.376	11 (8.1)	5 (3.7)	0.063	2 (1.5)	5 (3.7)	0.218
Cisplatin cycle									
<5 cycles, n=67	8 (11.9)	10 (14.9)	0.439	3 (4.5)	-	0.262	2 (3.0)	1 (1.5)	0.609
≥ 5 cycles, n=192	9 (4.7)	18 (9.4)	0.191	14 (7.3)	9 (4.7)	0.223	3 (1.6)	8 (4.2)	0.138
Cisplatin total dose									
<200 mg, n=52	6 (11.5)	12 (23.1)	0.25	1 (1.9)	1 (1.9)	0.867	1 (1.9)	1 (1.9)	0.867
≥200 mg, n=207	11 (5.3)	16 (7.7)	0.243	16 (7.7)	8 (3.9)	0.107	4 (1.9)	8 (3.9)	0.202
Brachytherapy techniques									
2D Planning, n=171	12 (7.0)	19 (11.1)	0.154	13 (7.6)	9 (5.3)	0.377	2 (1.7)	8 (4.7)	0.061
3D Planning, n=88	5 (5.7)	9 (10.2)	0.283	4 (4.5)	-	0.064	3 (3.4)	1 (1.1)	0.284

%, of events in all patients in each arm

## Author Contribution Statement

Chokaew Tovanabutra (CT) and Tussawan Asakij (TA) contributed to protocol development, data collection, manuscript writing, revision and approval. Kanisa rongsriyam (KR) contributed to data collection, data verification and analysis, manuscript revision and approval. Siriwan Tangjitgamol (ST) contributed to protocol development, grant acquisition, data collection, data verification and analysis, manuscript writing, revision and approval. Ekkasit Tharavichitkul (ET) contributed to protocol development, organizing and planning of the study conduct, data collection, manuscript revision and approval. Jirasak Sukhaboon (SK), Lieutenant Col. Apiradee Kridakara (AK), and Kannika Paengchit contributed to protocol development, data collection, manuscript revision and approval. Jakkapan Khunnarong (JK), Thiti Atjimakul (TA), Piyawan Pariyawateekul (PP), Prapai Tanprasert (PT), and Tharatorn Tungkasamit (TT) contributed to data collection, manuscript revision and approval. Vicharn Lorvidhaya (VL) contributed to protocol development, organizing and planning of the study conduct, data collection, manuscript revision and approval. 
